# Experiences of school health nurses regarding the provision of the school health service delivery in the Tshwane district

**DOI:** 10.4102/phcfm.v10i1.1807

**Published:** 2018-11-19

**Authors:** Selebetswe T. Dibakwane, Mmapheko D. Peu

**Affiliations:** 1 Department of Nursing Science, University of Pretoria, South Africa

## Abstract

**Background:**

Irrespective of the provision of an integrated school health policy, the school health nurses continue to experience multiple challenges regarding the provisioning of school health service delivery.

**Aim:**

The aim of this paper was to explore and describe the experiences of school health nurses regarding school health service delivery in the Tshwane district.

**Setting:**

Schools in the Tshwane district in Pretoria were used in the study.

**Methods:**

A qualitative and descriptive phenomenological design was used to conduct the study. Purposive sampling techniques were used to select a sample from the population of school health nurses employed in the Tshwane district and conduct the enquiry because of their knowledge and experience of school health services. The researchers collected data by means of unstructured, one-on-one in-depth interviews. The Tesch data analysis method was used by the researcher and co-coder. The researcher identified categories, subcategories and themes and these were reduced into grouping topics that were related to one another.

**Results:**

Positive and negative experiences of school health nurses emerged. It was evident from the findings of the study that the factors affecting the quality of the integrated school health programme (ISHP) provided were interrelated. Most of these factors negatively affected service delivery.

**Conclusion:**

It was recommended that the partnership between the National Department of Health and National Department of Basic Education as the main role players should be sustained at all times to ensure the successful implementation of the ISHP.

## Introduction

Worldwide, school-based health promotion is promulgated by the policy- and law-makers governing school health services. Various countries including South Africa are guided by their own school health promotion policies in order to coordinate the school health services. These include among others, preventive, promotive, curative and rehabilitative services that are necessary for optimal health of learners. The school environment settings should aim at supporting and promoting the emotional and physical well-being of the learners. The school health nurses are health care professionals and key personnel of the integrated school health policy (ISHP) who play a pivotal role and are responsible for screening, diagnosing, treating and referring learners to referral units. Therefore, young learners should be taught by the school health nurses to invest into their healthy lifestyle to enhance their lifespan.^1^

In South Africa, it is mandatory to document the interventions that promote school health services.^2^ It is documented that the policy can be provided through developing and implementing comprehensive wellness policies that focus on the needs of all learners.^3^ It is the responsibility of school health nurses to keep themselves abreast of new information concerning policies and guidelines on school health services and ethically assess their decisions on implementing such developments.^2,4^

The South African ISHP is a coordinated health care service delivery programme, which aims to address the needs of learners, teachers and the community. The policy as a programme, jointly developed by different stakeholders, aims to address the health and developmental needs of communities in South Africa.^2^ The confirmation was made that since 2012, the ISHP is an integral part of primary health care (PHC).^5^ The authors further note that the PHC services should be accessible, available, affordable and equitable to learners from diverse cultures. The community should be allowed to participate in making sure that school health services are rendered as part of PHC within the communities where the schools are built. Several authors emphasise that community members and families should participate and be involved in school health activities.^2,3,6^ Comprehensive and multisectoral input from the stakeholders and the school health nurses is required to address the physical, social and psychological needs of learners, teachers and the community.

School health nurses are the health care providers who are expected to provide the initial and sustained interventions to learners in the school environment.^7^ The school health nurses are the key personnel to identify and address health challenges that learners face, including depression and other mental health problems.^8,9^ On the other hand, school health nurses are facing challenges such as shortage of staff, lack of facilities and lack of support by school management boards. All these issues impede on school health delivery processes.^2^ School health nurses have been facing challenges in execution of their responsibilities on the roll-out of the programmes. These nurses are expected to work in a conducive environment in order to optimally execute their duties guided by the scope of practice. For them to deliver quality, up-to-date and safe health care as health care providers successfully, they need to be efficient in research, counselling and collaboration.^10^

### Problem statement

There is a shortage of school health nurses that is compounded by poor infrastructure development, lack of resources, very little support from parents and lack of support from school management boards and other stakeholders’ support. The majority of schools were not visited in accordance with the ISHP. Few health screenings were conducted at few schools with no follow-up. These problems create gaps in rendering the ISHP to public and special schools thus leading to poor school health service delivery. The South African Constitution,^11^ together with the World Health Organization (WHO) endorses the implementation of school health services.^2^ Therefore, there was a need to explore and describe the experiences of school health nurses regarding school health service delivery in Tshwane district.

### Research question

What are the experiences of school health nurses regarding the provision of school health service delivery in Tshwane district?

### The aim of the study

The aim of the study is to investigate the experiences of school health nurses’ regarding the provisioning of school health service delivery in Tshwane district.

## Research design and methods

The study followed a qualitative, descriptive phenomenological and contextual approach^12^ in order to allow the researcher to gain more information on the phenomena.^13^ A descriptive phenomenology is a philosophy and as a design grounded within Husserl’s philosophy. Descriptive phenomenology was developed by Husserl in 1962 who was interested in description of human experience.^12^ The proponents of descriptive phenomenology emphasise careful description of usual conscious experiences of daily life.^12^ For the purpose of this study, the experiences of school health nurses regarding school health service delivery in the Tshwane district were explored and described.^12^ This design was directed by the four steps: bracketing, intuiting, describing and analysing. Intuition happened when researchers became open to the meaning brought to the phenomenon by those who experienced it.^12^ During data collection and analysis, the researcher was completely immersed through careful listening to the experiences as they evolved.

### Research setting

Participants’ natural setting was their offices at place of work, when some participants used the schools as their settings where interactions took place. The offices of the participants as well as the schools used were located in the Tshwane district in Pretoria. Pretoria is the second biggest city in Gauteng Province in South Africa. Gauteng Province is divided into five districts, namely, Tshwane and/or Pretoria, West Rand, Ekurhuleni, City of Johannesburg and Sedibeng. Tshwane district is further subdivided into seven sub-districts. In 2014 there were 550 primary schools, 193 secondary schools in Tshwane district as well as 20 special government schools. Since 1994, many changes have occurred in the education system, where the previously disadvantaged and advantaged were integrated.

### Population

The target population comprised 20 available and accessible female school health nurses who provided health care services in the Tshwane district.^12,14^ No men were involved because during the time of study there were no male nurses providing school health services. The ages of participants ranged between 25 to 50 years. The experience of participants was 1 year and above. They possessed Diploma in Nursing and Midwifery and some qualified with Diploma in Primary Health Care. These participants were involved in the provision of school health service delivery.

### Sampling

Purposeful sampling was used in this study to select 20 school health nurses. The participants had more than 1 year experience in the delivery of school health services in the Tshwane district. In this study, the school health nurses were identified because they had relevant, first-hand experience and information related to school health service delivery.^12,14^

### Data collection

Data were collected through the use of unstructured one-on-one in-depth interviews with the support of field notes and audio recorder. Data were collected until data saturation was achieved. The one-on-one interviews allowed the researcher to observe nonverbal cues such as body language, gestures and the participants’ facial expressions. On the day of the interview, the researcher welcomed the participants and introductions were made to ease any tension. The research question, ‘What are the experiences of school health nurses regarding school health service delivery in Tshwane district?’, was posed. The following communication skills and strategies were used: probing, reflecting, clarifying, paraphrasing and listening. These skills were maintained to elicit more information. After every interview, the researcher thanked the participants for their time and contribution.

### Data analysis

Tesch’s data analysis method (1990) was used.^15^ The researcher carefully read through all the transcripts and identified concurrent themes. The researcher selected the document richest in data and studied it to ascertain the underlying meaning. Then, the researcher compiled a list of all the themes and grouped similar topics together. Columns were made, and data were sorted according to major categories, subcategories and emerging themes. This list of themes was abbreviated into codes and transferred next to the appropriate segments of text. This was done to ensure that all categories and codes were covered. Themes relating to one another were grouped together. The researcher also associated categories with each other by drawing lines to indicate interrelationships. All the material was then assembled into one document and categories coded where necessary. All the data from data collection were backed-up to ensure credibility.

### Trustworthiness

Trustworthiness was established through credibility, dependability, confirmability and authenticity.^12^ The credibility of the study was ensured by engaging in activities that produced confidence in the truth of the data. The researcher involved the co-coder during data analysis to ensure credibility of the results because the co-coder’s interventions reduced the risks of being biased.^12^ In ensuring prolonged engagement, the researcher spent sufficient time with participants; which was 3–6 months. The researcher was able to build a trusting relationship in order to obtain accurate and rich information.^16^ The findings from the analysed data were taken back to the participants to verify if data were accurate. To enhance the credibility of results, the researcher was supervised by an experienced qualitative researcher. The researcher also provided sufficient and descriptive data of the findings.

### Ethical considerations

Approval from the Research Ethics Committee of the University of Pretoria was obtained before data collection. (reference no. 443/2013)

## Results

Two categories emerged, such as negative and positive experiences of the school health nurses regarding provision of school health services. These categories were supported by the subcategories and themes (see Table 1).

**TABLE 1:** Categories, subcategories and emerging themes on the experiences of the school health nurses.

Categories	Subcategories	Themes
Positive experiences	1.1 Integrated School Health Programme	Appreciation of the services of school health nurses Benefit to learners Comprehensive health care
	1.2 Primary health care services	On-site treatment for learners
Negative experiences	2.1 School and health care facility infrastructure	Lack of space No proper offices for school health nurses Vehicles and shacks used as offices
	2.2 Staff shortages leading to increased workload	Increased numbers of learners
	2.3. Lack of management support	Insufficient support from management

### Positive experiences

Positive experiences focused on the ISHP and PHC services.

### Integrated school health programme

The ISHP included various programmes that assist school health nurses to provide health services. The study highlights that a full package of integrated school health programme (ISHP) as informed by the integrated school health policy (ISHP) ensures that the learners as recipients of health care benefit comprehensively from this service. Full involvement of learners, educators, parents and other stakeholders was ensured at all the time to enable learners to benefit from the initiative.

### Appreciation of the services of school health nurses

The participants noted positive satisfactory feelings when providing school health services to all learners. They emphasised that they are appreciated by learners, teachers and governing bodies. This was expressed as follows:

‘We are appreciated and seen as a necessity to the learners in the community because we are providing school health and primary health care package. The package is integrated and includes mother and child health services stakeholder support, health education, counselling for HIV & AIDS, TB and PHC reengineering. The package also assists us to address variety of problems such as malnutrition, dental carries, orphans, grants, abuse, disabled children, and barriers to learning.’ (Participant 15, female, 36 years, registered nurse)

‘I was well orientated to the school health services programme; it is something that is really valuable and is a good programme because it prevent diseases and promote health.’ (Participant 13, female, 36 years, registered nurse)

Another participant pointed out her experience as follows:

‘School health service delivery is a very important service that we need to deliver to our communities.’ (Participant 15, female 37 years, registered nurse)

The participants emphasised the importance of the school health services and indicated that it was not only appreciated by the nurses but also by the entire school community. The teachers found it convenient as they could easily refer learners with health problems to the school health nurses.

### Benefit to learners

This was explained as follows:

‘Our scope of practice is to do health screening, visual and hearing screening for learners to benefit. We identify visual impairments from learners and refer them to the optometrist for further screening, diagnoses and supplying them with spectacles if needed. The learners benefit a lot for receiving such services.’ (Participant 14, female, 36 years, registered nurse)

‘Children here are privileged to be having the service because we are identifying all those who have learning problems.’ (Participant 10, female, 40 years, registered nurse)

The participants indicated that the learners, teachers and parents needed the programmes in the ISHP because the parents were not even aware of the health care needs of their children. Therefore, inclusive ISHP is necessary.

### Comprehensive health care

The participants confirmed that the learners in the Tshwane district enjoyed the benefits of the ISHP because it is comprehensive in nature. Comprehensive health care means that when you visit school you offer all the services needed. Therefore, ISHP as stated by the Department of Health and Department of Basic Education is comprehensive and compulsory to all the schools. This policy encompasses variety of programmes in promoting the health of learners. The benefits of the ISHP were expressed by the school health nurses as follows:

‘We conduct comprehensive health care services as well as screening for hearing to prevent hearing loss. If we find any child with a hearing defect we refer them to the audiologist for further examinations. If any problem exists the learner is provided with hearing-aids. Furthermore, we provide immunizations particularly the catch-up immunizations for learners who were not immunized. We also conduct oral and dental health screening with dentists to detect early tooth decay. We administer deworming medicines and vitamin A in the treatment of acute skin condition and intestinal worms.’ (Participant 8, female, 41 years, registered nurse)

The participants reported that besides giving the relevant health education to learners, they also administer de-worming medication and vitamin A in the treatment of acute skin conditions, intestinal worms and improving vision. This indicates that the ISHP is comprehensive in terms of consultation on variety of conditions.

### Primary health care

The participants emphasised the importance of PHC particularly regarding the provision of on-site treatment for minor ailments. On-site treatment is the same as mobile service and it is part of the package of PHC.

### On-site treatment for learners

The participants indicated that learners, educators and parents were satisfied with the availability of on-site treatment.

‘We use three mobile vehicles at the clinics. Mobile cars are used for dental health care and eye care within for primary health care. Learners receive treatment on-site and do not have to go to the clinic for consultation.’ (Participant: 17, female, 33 years, registered nurse)

The participants explained that the three mobile vehicle clinics allocated to offer on-site treatment were a great benefit to both the school health nurses and the learners:

‘With the new integrated school health policy (ISHP) the treatment is offered on-site through the primary health care (PHC) mobile trucks. At the clinics the learner had to wait for hours in queue to be given treatment.’ (Participant: 7, female, 44 years, registered nurse)

The participants stated that the availability of three mobile vehicle clinics in schools was appreciated for assisting with early case findings.

### Negative experiences

Negative experiences focused on the school and health care facility infrastructure, staff shortages leading to increased workload and lack of management support.

### School and health care facility infrastructure

School and health care facility infrastructure addressed a lack of space, no proper offices for school health nurses, and vehicles and shacks (informal house) used as offices.

### Lack of space

The participants emphasised that there was lack of space to conduct adequate health screenings, no lights or tap water and the schools were using pit latrines as toilets. The lack of proper health care infrastructures had a negative impact on both the school and the school health service delivery.

The participants described the infrastructure challenges as follows:

‘The schools differ, but all I can say is that the majority of schools are receptive to us. The only challenge is that in most schools we do not have adequate or enough space where we can work. Sometimes we have to use classrooms.’ (Participant 6, female, 43 years, registered nurse)

‘Some schools do not have electricity, no adequate circulation of fresh air and sometimes you cannot even open the windows. Some schools are also still using pit latrines.’ (Participant 14, female, 36 years, registered nurse)

The participants explained that sometimes they were forced to improvise with regard to space by using their vehicles or squeezing into a shack to perform health assessments and screening. This caused a lot of inconvenience and affected the quality of their work:

‘Sometimes we screen learners in the staff or computer room with several disturbances. At some stage we are provided with a small room because old buildings were built without consideration of the school health nurses services.’ (Participant 7, female, 44 years, registered nurse)

‘I was hired without an office and I was using a wendy house. It is pathetic because you eat inside a shack? The wendy house is too small to fit nine school health nurses.’ (Participant 12, female, 38 years, registered nurse)

### No proper offices for school health nurses

The negative experience pertaining to office space was described as follows:

‘We are reporting to the local clinics and Community Health Centre (CHC). Most of us have no working space. Presently I am working from a garage.’ (Participant 1, female, 55 years, registered nurse)

Another participant reported on the poor school and health care facility infrastructure problems as follows:

‘The farm schools have no space to conduct some hearing tests. I need a very quiet place not mobile classes but it is mobile classes and they are not sound proof.‘ (Participant 2, female, 52 years, registered nurse)

### Vehicles and shacks used as offices

The participants characterised their working space as inadequate for meetings and keeping up with administrative functions such as statistics. This presented itself as a big inconvenience:

‘We do not have offices. The clinic is supposed to be our office, there must be an office for daily activities. I am currently do not have a car to do my administrative work.’ (Participant 12, female, 38 years, registered nurse)

The participants indicated that the old school buildings were small and had no allowance to accommodate them. In most cases, the school improvised space to allow them to render school health services.

### Staff shortages leading to increased workload

In this subcategory, the following theme emerged: increased numbers of learners.

#### Increased numbers of learners

The participants explained that the school health care team consisted of a professional nurse and a staff nurse or nursing assistant. Learner numbers seemed to increase, because of not only staff shortages but also parents’ inability to take learners to the clinic themselves. This was elaborated as follows:

‘We need more manpower. According to the integrated school health policy, the ratio of health care professional to learner should be 1:2000 per year; but at the moment I have to see 6000 learners. Sometimes I am unable to finish my allocated schools.’ (Participant 12, female, 38 years, registered nurse)

‘I have 27 schools to service.’ (Participant, 3, female, 49 years, registered nurse)

‘Parents do not take their children for immunization; so most of the time we are doing immunization where we should be assessing children.’ (Participant 6, female, 43 years, registered nurse)

The participants observed large numbers of learners registering at schools in January 2014, but the number of the school health teams remained the same. They also noticed the number of new public schools was on the increase, thus increasing their workload as well. What they were concerned about was that there were no replacements for the retiring staff and those on study leave.

#### Lack of management support

Leadership and management were essential in guiding and supporting an organisational goal. Lack of management support was verbalised in line with the insufficient support from management.

#### Insufficient support from management

The participants expressed their dissatisfaction with the lack of sufficient support from management:

‘We are not managing at all; we end up doing an injustice to kids, honestly speaking, at the end of the term. You only see statistics, not quality.’ (Participant, 3, female, 49 years, registered nurse)

‘Managers are giving us a little bit of support. We need them to come and experience ground level service from time to time. We are currently working in rural areas without an allowance. We know that other health care professionals are receiving an allowance.’ (Participant 6, female, 43 years, registered nurse)

## Discussion of results

### Positive experiences

Positive experiences were found to be related to the ISHP and PHC services. It was evident that the school health nurses who were providing school health services in the Tshwane district experienced not only negative work environments but also positive work environments. ISHP and PHC came up very strongly under positive experiences from the participants. It was noted that the ISHP serves as a guide for the school health nurses in the provisioning of school-based health promotions.^2^ It was confirmed that school-based health promotions are a legal requirement as a guide for the school health nurses in the provision of school health services.^12,17^ The school health nurses were responsible for conducting visual and basic hearing tests, measurement of height, weight and body mass index (BMI), checking for fine and gross motor skills problems, conducting oral health checks and screening for chronic illnesses or long-term health conditions such as tuberculosis and HIV and/or AIDS.^2^ This is confirmed by the Department of Health and Basic Education,^2^ which emphasises that school health nurses are also required to perform various tests including basic mental health or psychological risk assessment including mouth, dental and ear screening.

In this study, it was reported that because the parents were generally not taking their children to clinics for health-related issues, the ISHP played a crucial role in the learners receiving comprehensive health care services such as on-site treatment, dental care and eye care, which are basic human rights for South Africans.^2^ It was emphasised that ISHP is a mechanism in place to ensure that from 2015 onwards, learners are able to complete their primary education without absences from school because of illnesses that could have been screened and possibly prevented through this programme.^18^

The Department of Health^2^ confirms that ISHP is a component of PHC and it is protected by legislation and policy in a programmatic context as a basic right of all learners.^2^ It was further documented that the PHC service package entails that the service should be accessible, available, affordable, equitable, effective and efficient to all learners in order to be successful.^8^ Therefore, the participants noted that the goal of PHC was to enable and empower the community to attain the greatest possible biological, psychological and social well-being.

It was revealed in this study that health promotion should be conducted to prevent possible diseases. The NHP states that the promotion of good health and prevention of diseases is central to the success of PHC. This PHC should be sustained in order to achieve the Alma-Ata declaration commitment made by the world leaders in 1978 at Alma-Ata.^13^ School health service delivery during on-site visit was seen as an important activity to the learners because dental and oral care was offered through mobile clinic vehicles. PHC advocates an approach to health care that is based on the principle that all citizens have the right to receive accessible, affordable, efficient and effective care through their full participation in PHC settings.^3,5^ The community should be able to preserve their own health through empowerment and thus be able to increase access to the understanding and use of information in a method which maintains and promotes good health.^8,12^

A multidisciplinary team approach was expressed as a crucial approach to facilitate the school health service delivery as confirmed by Wainwright.^19^ Dennill^5^ explain that a truly multidisciplinary health team must be available in order to achieve effective intersectional action in health care provision. For the ISHP to be successful as a component of PHC, partnerships between education and health sectors, teachers, health workers, schools, community groups and the learners should be nurtured.^2^ The implementation of the ISHP was seen as a great benefit to learners who experience hearing, vision and other major health concerns. School health nurses, in addition, also offer comprehensive holistic health care solutions for mental health and substance-abuse issues, intervening by breaking the social isolation associated with these and other conditions.^14^ These findings were confirmed by Heflinger and Christens^20^ and point out the same sentiment in a similar study. It was found that the school health nurses provide physical, social, emotional and academic support to the learners. The presence of school health nurses in schools decreases absenteeism, especially learners with asthma and other conditions. The researchers are of the opinion that there is a need for multidisciplinary team approach to facilitate school health service delivery.

### Negative experiences

In this study, the participants emphasised that negative experiences were articulated as hampering school health service delivery. These included the lack of transport and shortage of staff, poor infrastructure of schools and health care institutions. The Constitution of South Africa documents that all the citizens have the right to health care that meets the principles of Batho Pele and/or People First.^21^ The study participants strongly affirm that all individuals should receive care according to Batho Pele principles. The *Patients’ Rights Charter*, in addition, regulates that every patient has a right to a healthy and safe environment.^21^ Additionally, in the new ISHP report, it was indicated that poor school and health care facility infrastructure had a negative impact on school health service provision delivery. The South African Government has also pledged the notion that puts children first.^21^ Furthermore, the report acknowledged that inequitable distribution of resources in both urban and rural settings contributed to the suboptimal school health care services.

The researchers discovered that the lack of resources and poor quality of the equipment used by the school health nurses compromised the quality of care. The participants expressed that sometimes they used their own money to buy batteries for ear, nose and throat sets in order to examine the learners’ ears, nose and throat.^8^ It was confirmed that the lack of resources was regarded as one of the constraints the school health nurses face in delivering school health services.^22^ Various authors^7,22^ agree that the school health nurses express that there should be enough space allocated for eye, hearing and BMI screening in order to properly perform these school health services. In addition to the much-needed space, the school health nurses identify and implement many health-related activities for learners with special needs.^23^ ‘Special needs of children in the public schools’ were identified as some of the challenges the schools are facing with special needs children.^24^ These needs may only be addressed by adequately prepared health care professionals in the public school settings. Moreover, school and facility infrastructure play an important part in the provision of effective and efficient school health care services to learners, especially those with special needs.

Negative experiences in the form of a shortage of nurses came up very strongly. The participants in this study argued that when some nurses retire or go on study leave or resign there are no form of replacement, thus increasing further shortages. Furthermore, these participants were of the opinion that they could not conduct follow-up visits and home visits because of staff shortages and time constraints. The findings of this study confirmed that inadequate staff threatens the quality of care to learners.^7^ To address the negative experiences of staff shortages, which impacts on school health service delivery, the government highlighted its commitment to employing additional staff or retired nurses as additional resources.^2^ In confirmation,^25^ it was noted that there is a need to allocate sufficient staff to equally distribute good quality health care in order to reduce staff shortages.

The participants in this study repeatedly mentioned that managers fail to support them during difficult times. The participants revealed that they lack the support of their managers because some schools had no electricity, no running water and no toilets. The participants noted that the support, commitment and regular visits by personnel on behalf of management may contribute positively towards the delivery of quality school health services. The concerns the school health nurses had was that school health service constraints should be addressed in time. A lack of leadership support is a barrier to effective school health service delivery.^26^ Therefore, the authors are of the opinion that there is a need, by management, to support the school health nurses.

### Implications

The most important implication of this study involves the ISHP service implementation. It was stipulated that no learner will be deprived of the quality school health care as documented in the South African Constitution, the *Bill of Rights* and the *National Health Act*. Special attention must be paid to the school health nurses needs in order to facilitate the delivery of quality school health services.

### Limitations of the study

The study was limited only to female school health nurses and therefore findings report only limited important experiences of male school health nurses, teachers, learners and school governing body. It was observed that the ISHP coverage was not equally implemented in other provinces as mandated by the government policy document at the time.

## Conclusion and recommendations

The findings of this study highlighted both positive and negative experiences of the school health nurses regarding provision of school health service delivery. Emphasis was put on the ISHP and PHC representing positive experiences. The importance of ISHP as a package is a necessity for health promoting school and provision of PHC. On the other hand, negative experiences focused on poor school and health care infrastructure such as lack of space, lack of offices, lack of vehicles, increased number of learners and insufficient support when providing school health service delivery suggest that the school health nurses play a vital role in the delivery of school health services in schools, provided that adequate facilities are available. The experiences of school health nurses were identified, and possible solutions were suggested. It can be concluded that the objective of the study has been well achieved. It is believed and hoped that corrective action will be taken in order to improve school health service delivery. Based on the findings of this study, it was recommended that all governments are mandated to cooperate and innovate national policies. There must be action plans to launch and sustain the reengineering of primary health through collaboration between and across involved sectors and departments. It is important also to conduct future studies including female and male school health nurses, teachers, learners and school governing bodies to elicit comprehensive results.
